# Artificial Intelligence (AI) in Nuclear Medicine: Is a Friend Not Foe

**DOI:** 10.1055/s-0043-1777698

**Published:** 2024-01-22

**Authors:** Maseeh uz Zaman, Nosheen Fatima

**Affiliations:** 1 Department of Radiology, Aga Khan University Hospital, Karachi, Pakistan

Artificial intelligence (AI) is the most robust and fast-growing entity in current era that will revolutionize almost every corner of human lives. The term AI was first coined during a summer workshop at Dartmouth College, United States in 1956. 1 But, AI in current days has captured a front seat thanks to immense development in computing power, storage, advanced algorithm, and huge database. AI refers to an arena of computer science focused at replicating the performance of tasks typically requiring human intelligence. 2 Briefly humongous AI progress has been in the order of machine learning (ML), deep learning (DL), artificial neural networks (ANNs), convolutional neural networks (CNNs), and generative adversarial networks (GANs). 3 Discussing these entities is beyond the scope of this editorial.

Precision medicine is aimed to develop tailor-made diagnostic and therapeutic approaches for individual patients. Integration of AI plays a significant role in achieving precision medicine in nuclear medicine (NM) and molecular imaging. AI has multifaceted applications in NM and molecular imaging including image planning, acquisition, processing, interpretation, diagnosis, prognosis, and prediction of response to therapy. On administrative side, it can also be used for patient admission and payments. 4 AI assimilated into patients' appointment and triage algorithms can significantly rationalize appointments, self/rescheduling and also prioritizing some visits. 5 For outpatients, AI has the potential to enable patients to determine a location and time that work best for them. Similarly, in admitted patient setting, AI algorithm can automatically prioritize certain NM examinations with the goal of optimizing patient care. 6 AI can also assess justification for requested procedure, contraindications based on history of allergies, and drug interference and also avoiding an unjustified repetition by evaluating prior studies conducted on same patient. 7 Daily quality control (QC) of NM scanners is important to ensure a scanner is properly functioning and in case of error it permits timely service calls to be placed, ensuring operational integrity and quality imaging. QC of NM/PET (positron emission tomography) scanners can take as little as 30 minutes or as long as 3 to 4 hours and nonavailability of imaging services. AI-based scanner acquires the QC data during off-hours and stores the information in the acquisition terminal for review and acceptance by the technologist that save precious time. So, AI-based QC systems can significantly improve the efficiency of NM technologists and departments throughput. 8 In future, AI would be integrated into patient verification algorithm at each step and smart syringe would allow automatic selection of radiopharmaceutical and radiopharmaceutical dose to be administered that can significantly reduce the chances of human errors. 9 After administration of radiopharmaceutical, many PET and NM studies require patients to wait in a secluded waiting environment for a certain period that is definitely a source of anxiety in some vulnerable patients. To mitigate patients' anxiety, emotionally intelligent AI-based devices like Smart Chatbots having a kind and loving temper to mimic the best of human empathy may be deployed. 10

One of important responsibilities of NM technologists is to select the correct scanning protocol depending on the prescribed NM test by the nuclear physician. Most of picture archiving and communication systems have integrated AI into protocol selection that can improve workflow efficiency by using available pertinent clinical information in the electronic health record. 11 AI can also be used to optimize contrast volume, injection rates based on patient characteristics, and selection of current field-of-view (FOV) based on information about location of lesion in previous study. So, AI can alert the NM technologist to ensure whether a particular area is included in current FOV or excluded by an educated decision making.

During acquisition of NM and PET imaging, accelerating scanning time and reducing radiation dosage are promising aspects. In future, AI-based real-time image quality assessment evaluation may become a critical component of patient quality and safety data. 7 So, by adopting this methodology, technologists would be notified of image quality issues that would be corrected either by reacquisition or postacquisition data error correction. Certainly, this approach will decrease the need for patient recall that in NM has significant financial cost and unjustified radiation exposure. 7

AI can play an important role in processing of reconstructed NM and PET image for reducing noise, improving spatial resolution and extraction of image biomarkers for response evaluation in serial imaging. Various researchers have used large pixelated crystal with CNN to reduce noise and improve image resolution. 12 Another group has shown that use of denoising CNN in iterative PET reconstruction with local linear fitting has significantly reduced noise and improved image quality. 13 In NM and PET image processing, attenuation maps and scatter correction are one of topics for research. Hwang et al generated attenuation maps for whole-body positron emission tomography/magnetic resonance imaging (PET/MRI) using a specialized convolutional network architecture for image segmentation. 6 They have compared the CT-derived attenuation map to the Dixon-based 4-segment technique. 6

Prima facie, AI seems a threat to the role of the NM technologist; however, by automating mundane chore using AI, they can revisit their role as supervisor, overseeing AI-based QC and ensuring effective communication with patients. It is important that medical imaging professional societies and colleges should constitute task force to formulate and implement pertinent frameworks and guidelines. These guidelines must include basic standards for use and validation of specific AI system. Furthermore, these task forces and committees (having NM technologists as members) should assist policymakers in drafting legislations for the safe use of AI. 14 In NM and molecular imaging, meaningful implementation of AI needs educational programs targeting its scientific and clinical aspects. These academic activities will abreast current and future clinicians and technologists with AI that would enhance its potential adoption and development.

We are living in the era of AI and NM and molecular imaging is not an exception. AI has great potential in image planning, acquisition, processing, interpretation, diagnosis, prognosis, and prediction of response to therapy. In NM and molecular imaging, AI also has the potential to improve clinical workflows that will increase overall efficiency and also enable personalized medicine for better patient care. For clinical adoption, continuous robust multicenter and multigroup research and validation of their outcomes are of paramount importance. Effective and meaningful implementation of AI needs educational programs targeting the scientific and clinical aspects in NM and molecular imaging.
